# How do you feel right now? Investigating momentary well-being in French adolescents using geographically-explicit ecological momentary assessment

**DOI:** 10.1016/j.ssmph.2026.101956

**Published:** 2026-08-12

**Authors:** Marie Buzzi, Nelly Agrinier, Benoît Lalloué, Emeline Lequy, Fabienne Ligier, Jennifer O'Loughlin, Yan Kestens, Laetitia Minary

**Affiliations:** a CHRU-Nancy, INSERM, Université de Lorraine, CIC, Epidémiologie Clinique, Nancy, F-54000, France; b Université de Lorraine, Inserm, INSPIIRE, Nancy, F-54000, France; c Université Paris-Saclay, UVSQ, Université Paris-Sud, Centre for Research in Epidemiology and Population Health (CESP), 'Exposome and Heredity' Team, Institut pour la Santé et la Recherche Médicale (INSERM), Gustave Roussy, Villejuif, France; d Centre de Recherche en Santé Publique (CReSP), Montréal, QC, Canada; e École de Santé Publique de l'Université de Montréal (ESPUM), Montréal, QC, Canada

**Keywords:** Geographically-explicit ecological momentary assessment, Mental health, Well-being, Adolescence

## Abstract

**Introduction:**

Adolescence is a period of heightened emotional reactivity and vulnerability to mental health problems, during which well-being plays a central role in psychological adjustment. Beyond reflecting immediate experiences, momentary well-being has been linked to long-term outcomes such as depression and substance use. While social and geographic contexts are recognized determinants of adolescents’ mental health, their influence on momentary well-being remains unclear. We aimed to investigate these associations in a sample of French adolescents using real-time assessments.

**Methods:**

Participants were adolescents from the EXIST cohort, who agreed to participate in the Geographically-explicit Ecological Momentary Assessment (GEMA) sub-study. Over 10 days, participants received two SMS prompts daily linked to a short questionnaire. Geolocation was recorded at each questionnaire completion. We used mixed-effects linear regression models to assess the associations between momentary contextual factors and well-being.

**Results:**

A total of 122 adolescents provided 1236 observations. The median age was 13.4 years (IQR: 13.3-13.6). Being with friends or family was associated with higher momentary well-being compared to being alone (adjusted Beta (95%CI) = 3.5 (1.1-5.9), and 3.0 (1.6-4.5), respectively). There was a trend suggestive of an association between momentary well-being and exposure to green space, as measured by the average NDVI (Normalized Difference Vegetation Index) within a 100m radius. Girls and gender-diverse adolescents reported significantly lower levels of momentary well-being than boys.

**Conclusion:**

Immediate social context emerged as one of the strongest correlates of adolescents’ momentary well-being. Our findings suggest that supportive social relationships are closely associated with higher momentary well-being among adolescents. They also highlight the potential of GEMA for investigating contextual correlates of well-being and for informing future research on context-sensitive approaches to mental health promotion.

## Highlights

• Social context is strongly associated with adolescents' momentary well-being.
• Adolescents' well-being fluctuates with geographic features of their environment.
• GEMA can inform personalized and context-responsive mental health interventions.

**Implications and contributions**

Using Geographically-explicit Ecological Momentary Assessment, this study suggests real-time associations between social and geographical environments and momentary well-being among adolescents. It underscores possible value in context-sensitive approaches in developing real-time, adaptive interventions to promote youth mental health.

## 1 Introduction

Well-being plays a crucial role in adolescent health (Patton et al., 2016). Rather than simply an absence of psychological distress, well-being encompasses positive mental health including affective, cognitive, and social dimensions. Its association with a variety of health outcomes in adolescents including substance use, has been demonstrated in cross-sectional and longitudinal studies (Howell et al., 2007; Mytton et al., 2024; National Academies of Sciences E, Division H and M, Education D of B and Ss, 2019).

However, adolescence is also a time of profound transformation, characterized by heightened emotional reactivity and the ongoing development of emotion regulation skills (Ahmed et al., 2015). Together, these contribute to increased impulsivity and risk-taking behaviors. In this context, adolescents' well-being is likely to be influenced by daily fluctuations in both social experiences and the type of physical environment to which they are exposed (Silk et al., 2003; Weinstein et al., 2006). Importantly, growing evidence suggests that momentary well-being is not merely a transient state, but is linked to more enduring aspects of mental health, such as depression and anxiety, as well as a greater likelihood of engaging in health-risk behaviors (Silk et al., 2003). Thus, understanding short-term variations in well-being, and identifying the social and geographic factors influencing them, could provide valuable insights into mechanisms that contribute to long-term outcomes, and help identify “just-in-time” levers for intervention supporting adolescents’ mental health.

Ecological Momentary Assessment (EMA) (i.e., the repeated, real-time collection of data on individuals’ experiences in their natural environment (Shiffman et al., 2008)), offers unique methodological advantages for tracking such dynamic changes. When paired with passive geolocation tracking as part of Geographically-explicit EMA (GEMA)(Kirchner & Shiffman, 2016), this approach makes it possible to accurately document social and geographic exposures and their association with momentary well-being.

In recent years, a growing body of GEMA research has identified a range of contextual factors influencing momentary well-being, including weather conditions and sun exposure (Mascherek et al., 2025), proximity to natural or leisure environments (Bakolis et al., 2018; Beute et al., 2018; Mascherek et al., 2025), social setting (Lipperman-Kreda et al., 2017; Rusby et al., 2013), feeling of boredom (Zawadzki et al., 2015), neighborhood socioeconomic deprivation (Christensen et al., 2025), and violence (Gasik et al., 2025). However, two major limitations remain. First, although EMA research has increasingly been applied to adolescent mental health (Russell & Gajos, 2020), much of the GEMA literature investigating contextual correlates of momentary well-being has been conducted in adult populations. Given developmental specificities during adolescence, which may influence the associations between contextual factors and emotional experiences, the extent to which findings among adults generalize to adolescents remains unclear. Second, there is considerable heterogeneity in the conceptualization and measurement of momentary mental health. Extant studies have employed a wide range of overlapping constructs and most rely on ad hoc items or adaptations of retrospective measurement scales not validated for momentary data collection (Votaw & Witkiewitz, 2021). This compromises the psychometric validity of findings, and limits the comparability and interpretability of results across studies.

The aim of this study was therefore to examine whether associations similar to those observed in adults could be observed among adolescents, using a GEMA approach and valid measurement of momentary well-being in a sample of French secondary school students participating in the EXIST study (EXIST. Accessed July 6).

We hypothesized that, as observed in adults, being with friends or family members is positively associated with adolescents’ momentary well-being compared to being alone, that greater exposure to green space or sunlight is associated with higher levels of momentary well-being, and finally that living in a socioeconomically deprived area or an area with a higher crime rate is associated with lower well-being.

## 2 Methods

### 2.1 Setting: EXIST study

The EXIST study *(Explorer les mécanismes des inégalités sociales et territoriales liées au tabagisme et autres comportements à risque pour la santé – Explore the mechanisms of social and territorial inequalities related to smoking and other risky health behaviors)* is an ongoing longitudinal cohort study following adolescents for three years from eighth grade (age 13.5 (SD = 0.4) at inclusion). EXIST initially aimed to describe how individual and environmental characteristics evolve and interact over time among adolescents, and how these factors and contexts contribute to social inequalities in smoking by age 16, but its design and collected data allow for a broader research scope.

The project included 5491 adolescents from 53 schools in the Grand Est region of France. Schools were selected using a stratified sampling approach, from among 493 eligible public schools (each with ≥30 eighth grade students per class) in 2022. Strata were determined based on three criteria: regional education authority (Nancy-Metz, Reims, Strasbourg), classification as a priority education zone (no priority education, priority education, priority education plus) and geographic location of the school (urban, intermediary, rural). The cross-classification of the three criteria produced 27 possible strata, of which 17 contained eligible schools. A sampling fraction of 10% was applied within each stratum, via a computer-generated randomization list. The principal of each selected school was contacted to obtain agreement to participate in the study. If the response was negative, the next school in the same stratum was contacted, until all sampling slots were filled. For logistical reasons, enrollment took place in two phases: the first phase (2022–2023, N = 1414 adolescents) included fifteen schools: 4 pilot schools (N = 419 adolescents), selected pragmatically to ensure diversity in geographic location and socioeconomic characteristics, and used to refine the questionnaires content, and 11 schools (N = 995 adolescents) selected at random using the procedure described above. The second phase (2023–2024, N = 4077 adolescents) included 38 randomly selected schools.

Student-level data were collected three times a year in online self-administered questionnaires. In 8th and 9th grades (i.e., first two years of follow-up), data collection took place during school hours, replacing a 1-h class session. Students completed questionnaires using tablets and an online platform provided by Polygon (Polygon) with help from an on-site research team if needed. Questionnaires covered mental and physical health, health-related behaviors, and relationships with families, school, and peers.

Additionally, all participants were invited each year to participate in a 10-day GEMA sub-study on a voluntary basis, provided that signed consent was obtained from the adolescent and at least one parent. Participants were eligible for a raffle to win gift vouchers for a sports or cultural store.

This study is reported in accordance with STROBE-GEMA guidelines (Kingsbury et al., 2024) (Supplementary Material S1).

### 2.2 Sample

Participants in the present study were volunteers from the EXIST cohort. Both adolescents and their legal guardians received written information and provided informed consent for participation in the GEMA sub-study. Intensive data collection took place in Summer 2023 and Summer 2024, and eligibility required that adolescents own a smartphone.

### 2.3 Procedure

The GEMA protocol was conducted over ten consecutive days which sampled both week- and weekend-days (Myin-Germeys & Kuppens, 2022).

Because smartphones must be switched-off during school hours in most French secondary schools, SMS notifications were sent to participants twice a day according to a time-contingent design (de Vries et al., 2021): once in the morning and once in the evening (06:01AM and 05:01PM during week days, and 10:01AM and 05:01PM during weekend days). Participants had 180 min to begin completing the questionnaire after notification.

Each SMS included a link to a web application provided by Polygon (Polygon), where the questionnaire could be completed. Geolocation data were collected automatically using the smartphone location services when participants connected to the link. Successful geolocation required location services to be activated and the corresponding permissions granted by the participant. Because not all smartphone configurations allow automatic capture of GPS data, participants were previously instructed on how to provide authorization for geolocation data sharing on their smartphone, this authorization being optional.

At each assessment, participants were asked to report their momentary level of well-being using the Ecological Momentary Wellbeing Instrument (EMoWI), as well as their momentary level of boredom (on a scale from 0 [not bored at all] to 10 [very bored]), physical location (“At home”, “At school”, “Traveling”, “At a sports or leisure facility”, “In a park”, “At a friend's house”, “At a party”, or “Other”), and their immediate social setting (“I am alone”, “With a friend”, “With several friends”, “With my father or mother”, “With one of several family members”, “With persons I do not know”, “Other”). They were also asked whether they or the people around them had used tobacco, cannabis, e-cigarettes, and/or alcohol in the past 15 min.

### 2.4 Measures

#### 2.4.1 Baseline characteristics

Data on participants’ age, gender, family socioeconomic status, and personal history of chronic health conditions were extracted from the EXIST baseline questionnaire.

#### 2.4.2 EMoWI

The EMoWI is an 8-item pictorial well-being scale, designed for numeric use (Buzzi et al., 2022, 2025a). Each item measures a dimension of momentary well-being (i.e., self-confidence, stress, loneliness, happiness, energy, autonomy, safety, and feeling of being valued). It includes an instruction paragraph followed by a continuous line segment between two pictures representing the extremes of each dimension. For each item, participants move a slider on the line segment to the position that best represents their momentary experience, which is then interpreted as a discrete score ranging from 0 to 10. A global score is calculated by totaling the scores for all items, with reverse scoring of items measuring stress and loneliness. Higher scores represent higher levels of momentary well-being.

The psychometric properties of EMoWI were satisfactory in two preliminary cross-sectional assessments of both the French and English versions of the scale, conducted among samples of French (Buzzi et al., 2022) and Canadian adolescents (Buzzi, Patte, et al., 2025). In addition, the scale demonstrated satisfactory acceptability, dimensionality, reliability, and validity in an EMA study conducted among Canadian adults (Buzzi, Moullec, et al., 2025).

#### 2.4.3 Environmental characteristics

Environmental variables were derived from GPS coordinates recorded at the time of questionnaire completion.

Exposure to green space was assessed using the Normalized Difference Vegetation Index (NDVI), a widely used satellite-derived indicator of greenness, based on surface reflectance. NDVI values range from −1 to +1, with higher values indicating denser vegetation. We extracted NDVI data from the Landsat 32-Day NDVI Composite dataset, provided by the U.S. Geological Survey and hosted on Google Earth Engine (Landsat Collection 2 Tier 1 Level 2 32-Day NDVI and Composite). For each EMA record, NDVI values were extracted based on the participant's geolocation at the time the questionnaire was initiated, using data from the May-June 2024 period, which corresponds to the peak of the vegetation season in France. Negative NDVI values – indicative of clouds, water, or bare rock – were set to 0 before averaging within a 100-m buffer around the recorded geolocation. This buffer size has been validated as an appropriate scale for quantifying visible ground-level greenness in residential areas using NDVI data (Rhew et al., 2011).

Sun exposure was assessed using daily sunshine duration (in hours) recorded by the nearest weather station, determined by GPS proximity, on the day of each assessment. Data were extracted from the French national meteorological database *Météo-France*.

Two social indicators were used to characterize the area in which the adolescent was located at the time of questionnaire completion. A crime indicator was computed based on the rate of infractions (i.e., burglary, vandalism, armed robbery, and unarmed violent robbery) per 1000 inhabitants in the municipality in 2023 and 2024, according to the year of data collection, using official national statistics. Neighborhood social deprivation was assessed using the French Deprivation Index (FDEP), an area-level composite indicator developed to describe health inequalities (Rey et al., 2009). Neighborhoods were defined using IRIS units (i.e., aggregated units for statistical information – Ilôts regroupés pour l’Information Statistique), the smallest geographical division used by the French National Statistics Institute, with an average population of approximately 2000 residents per unit. The FDEP score is interpreted as a continuous measure, standardized to a mean of 0 across France, with higher values indicating greater deprivation and negative values reflecting more advantaged areas. FDEP scores were drawn from the most recent nationally available dataset (2015).

### 2.5 Analyses

Participants' characteristics were described. Characteristics of participants included in the analyses were compared to those of participants excluded because of missing data, as well as those of the adolescents from the EXIST cohort who did not participate in the GEMA sub-study. To assess the reliability of the EMoWI in the present sample, internal consistency was evaluated using MacDonald's ω.

The association between social and geographic factors and momentary well-being was assessed using a mixed-effects linear regression model with three hierarchical levels: prompt, day, and person. Consistent with recommendations for multilevel modeling, the random-effects structure was specified a priori to reflect the hierarchical organization of the GEMA data (Gelman & Hill, 2006). The number of observations and variance components were reported for each level. The global EMoWI score, assessed at each time point, was used as the dependent variable. Independent variables included person-level sociodemographic variables (i.e., age, gender, socioeconomic status, personal history of chronic health conditions), and prompt-level contextual variables (i.e., self-reported immediate location and social setting, boredom, green space, sunshine duration, social deprivation, and crime level). As participants could report being with multiple categories of people at the time of questionnaire completion, social-context categories were not mutually exclusive. Therefore, each category was modeled using a separate indicator variable, with being alone used as the reference category. Model assumptions were verified (i.e., normality of residuals using a QQ plot, homoscedasticity by a visual inspection of residual plots, and absence of multicollinearity by measuring Generalized Variance Inflation Factors (GVIFs) for all independent variables). Quantitative variables were included in the model in their continuous form, after checking the linearity of their relationship with well-being via visual inspection of plots and standardization, to enhance comparability and interpretability. We first estimated unadjusted models for each independent variable, followed by an adjusted multivariable model including all selected variables, and Nakagawa's R^2^ was used to evaluate model fit.

Sensitivity analyses were conducted to assess the robustness of findings to alternative modeling assumptions. First, analyses were repeated after excluding participants who identified outside the male-female binary, given the small size of this subgroup. Second, models were re-estimated after adding a school-level random effect to account for potential clustering within schools. Third, the main model was re-estimated without the boredom variable, given its conceptual proximity to momentary well-being.

Analyses were conducted on complete cases with a statistical significance level of 5%.

All analyses were conducted using R statistical software v4.4.1. R packages ‘terra’ (Hijmans et al., 2025) and ‘sf’ (Pebesma, 2018) were used for data management of spatial data.

### 2.6 Ethics

Prior to participating in EXIST, adolescents and their legal representatives were informed in writing about the study and their right to refuse to participate or to ask for removal of their data at any time.

The EXIST cohort and the GEMA sub-study were approved by the *Comité éthique et scientifique pour les recherches, les études et les évaluations dans le domaine de la santé* (CESREES), the *Commission Nationale de l’Informatique et des Libertés* (CNIL) (n°922169), and the *Inserm* IRB (i.e., the *Comité d’Evaluation Ethique de l’Inserm*) (CEEI) (n°21-837 and n°21-837 bis).

## 3 Results

**Fig. 1:**
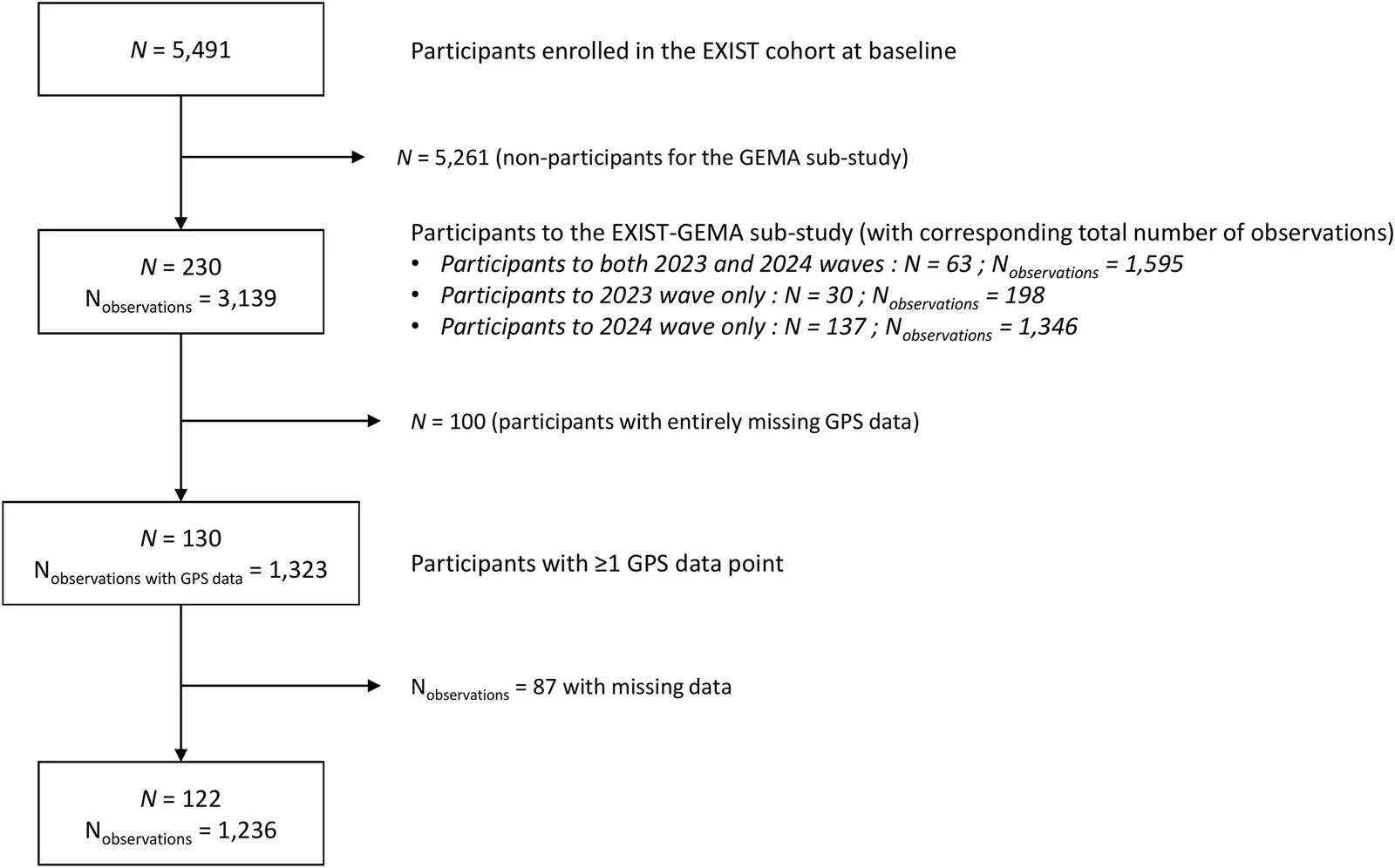
EXIST GEMA sub-study flowchart.

At baseline, 5491 adolescents were included in the EXIST study. Among them, 245 volunteered for the GEMA sub-study and 230 provided at least one complete questionnaire – 30 took part in the 2023 data collection wave only, 137 in the 2024 wave only, and 63 in both waves. Overall, these participants responded to a total of 3139 prompts, of a maximum of 4600 (68.2% response proportion) (Fig. 1).

Geolocation data were missing at all time points for 100 participants, preventing the use of their observations in spatial analyses, and 8 participants were missing data on factors potentially associated with well-being for all observations. Among the 122 remaining participants, 1236 observations had GPS data and were included in the final analytical sample.

Baseline characteristics of excluded participants were similar to those of included participants, except for a higher proportion of boys in the sample retained for analysis (Supplementary material S2). Compared to the EXIST cohort overall, the GEMA sub-sample included a substantially lower proportion of boys (51% vs. 32%) and a higher proportion of adolescents from very advantaged socioeconomic backgrounds (6.4% vs. 11%). Age and personal history of chronic health conditions were similar across groups (Supplementary material S3).

The characteristics of included adolescents are presented in Table 1. Two-thirds of participants were girls (63.1%); the median age of participants was 13.4 years (IQR: 13.3 – 13.6).

**Table 1:** Participants’ characteristics.

Characteristic	N = 122
Age (years)	
Median (Q1 - Q3)	13.4 (13.3 - 13.6)
Gender	
Boy	42 (34.4%)
Girl	77 (63.1%)
Other	3 (2.5%)
Socioeconomic status	
Very advantaged	14 (11.5%)
Advantaged	32 (26.2%)
Intermediate	26 (21.3%)
Modest	36 (29.5%)
Disadvantaged	0 (0%)
Unknown	14 (11.5%)
History of chronic health conditions	
No	102 (83.6%)
Yes	20 (16.4%)
Number of distinct location categories visited	
Median (Q1 - Q3)	2 (2 – 4)

Timing, location, geographic and social context at the time of GEMA questionnaire completion are detailed in Table 2. In the sample with GPS data, the median (IQR) number of completed questionnaires per participant over the study period was 9.0 (4.0-13.3) in 2023 and 6.5 (2.8-13.3) in 2024. Participants were more likely to respond to evening questionnaires (61% of observations). When completing the questionnaire, adolescents were most often at home (66.7%) or traveling (17.1%), and most commonly reported being with family (69.5%) or alone (31.6%). Participants reported a median of two distinct location categories (IQR = 2-4) during the 10-day study period. The mean (SD) EMoWI score across all responses was 62 (16). Variance component analysis showed that most variability in momentary well-being (50.9%) was attributable to differences between participants, whereas day-level variability accounted for 7.6% of the total variance (Supplementary Material S7). Consistent with previous validation studies, the EMoWI showed satisfactory internal consistency in the present sample (MacDonald's ω = 0.87), supporting use of a summed score.

**Table 2:** Characteristics of GEMA questionnaires completed by participants.

Characteristic	N = 1236
EMA data collection wave	
2023	395 (32%)
2024	841 (68%)
Day of the week	
Monday	129 (10.4%)
Tuesday	235 (19.0%)
Wednesday	224 (18.1%)
Thursday	234 (18.9%)
Friday	182 (14.7%)
Saturday	200 (16.2%)
Sunday	32 (2.6%)
Time of day	
Morning	482 (39.0%)
Evening	754 (61.0%)
Where are you?	
At home	825 (66.7%)
At school	46 (3.7%)
Traveling (e.g., at a bus stop, on a train, in a car, etc.)	211 (17.1%)
At a sports or leisure facility	33 (2.7%)
In a park	11 (0.9%)
At a friend's house	14 (1.1%)
At a party	12 (1.0%)
Other	84 (6.8%)
Average NDVI within a 100m radius	
Mean (SD)	0.57 (0.12)
Missing	7
French Deprivation Index	
Median (Q1 - Q3)	0.49 (−0.57 - 0.85)
Community crime level (‰)	
Median (Q1 - Q3)	2.4 (0.0 - 16.2)
Sunshine duration on measurement day (hours)	
Mean (SD)	8.4 (5.2)
EMoWI global score (range: 0-80)	
Mean (SD)	62 (16)
Momentary level of boredom (range: 0-10)	
Mean (SD)	2.9 (3.4)
Who are you with right now? (one or more answers possible)	
No one, I am alone	391 (31.6%)
A friend	111 (9.0%)
Several friends	116 (9.4%)
My father or mother	366 (29.9%)
One or several family members	363 (29.6%)
Persons I do not know	60 (4.9%)
Other	65 (5.3%)
Time to questionnaire completion (minutes)	

Median (Q1 - Q3)	30 (4 - 121)
Missing	23
Time of questionnaire completion (*weekday morning questionnaire*)	
Median	08:06
Missing	13
Time of questionnaire completion (*weekend day morning questionnaire*)	
Median	10:12
Missing	1
Time of questionnaire completion (*evening questionnaire*)	
Median	17:11
Missing	9

NDVI: Normalized Difference Vegetation Index.

Findings of the mixed-effects linear regression model (Table 3) indicate that adolescents' immediate social setting was significantly associated with their momentary well-being. Being at a friend's house was associated with higher well-being compared to being at home (adjusted Beta (aB) = 5.9, 95%CI: 0.25 to 12). Similarly, being with several friends or one or several family members other than parents was linked to higher well-being (aB = 3.5, 95%CI: 1.1 to 5.9 and aB = 3.0, 95%CI: 1.6 to 4.5, respectively), whereas being among unknown persons was associated with lower well-being (aB = −4.6, 95%CI: −7.7 to −1.5). Momentary boredom was negatively associated with well-being (Beta = −5.2, 95%CI: −5.8 to −4.5).

**Table 3:** Beta coefficients and 95% confidence intervals for the associations between contextual factors and momentary well-being in adolescents from the EXIST-GEMA sub-study.

Characteristic (N = 1229)	*Unadjusted model*	Adjusted model	Adjusted GVIF^b^
	Beta (95% CI)	Beta (95% CI)	
Where are you?			1.1
At home	—	—	
At school	−3.9 (−7.3 to −0.47)	−0.91 (−4.4 to 2.5)	
Traveling (e.g., at a bus stop, on a train, in a car, etc.)	−2.6 (−4.4 to −0.66)	−0.97 (−2.9 to 0.91)	
At a sports or leisure facility	1.0 (−3.0 to 5.0)	0.66 (−3.3 to 4.6)	
In a park	9.6 (2.8 to 16)	5.8 (−0.56 to 12)	
At a friend's house	10 (4.2 to 16)	**5.9 (0.25 to 12)**	
At a party	2.5 (−4.0 to 8.9)	0.25 (−5.8 to 6.3)	
Other	0.76 (−1.9 to 3.4)	−0.43 (−2.9 to 2.1)	
Average NDVI within a 100m radius^a^	0.63 (−0.22 to 1.5)	0.53 (−0.30 to 1.4)	1.1
French Deprivation Index^a^	−1.1 (−2.2 to 0.01)	**−1.2 (−2.2 to −0.17)**	1.0
Community crime level (‰)^a^	−1.3 (−2.4 to −0.18)	−0.20 (−1.3 to 0.89)	1.1
Sunshine duration on measurement day (hours)^a^	0.48 (−0.32 to 1.3)	**0.98 (0.21 to 1.7)**	1.1
Who are you with right now?			
*A friend*			1.1
No	—	—	
Yes	0.76 (−1.6 to 3.1)	1.2 (−1.0 to 3.4)	
*Several friends*			1.2
No	—	—	
Yes	3.6 (1.3 to 6.0)	**3.5 (1.1 to 5.9)**	
*My father or my mother*			1.0
No	—	—	
Yes	1.7 (0.25 to 3.2)	1.0 (−0.38 to 2.4)	
*One or several family members*			1.1
No	—	—	
Yes	4.3 (2.8 to 5.8)	**3.0 (1.6 to 4.5)**	
*Persons I do not know*			1.1
No	—	—	
Yes	−5.9 (−9.1 to −2.6)	**−4.6 (−7.7 to −1.5)**	
*Other*			1.0
No	—	—	
Yes	−1.4 (−4.5 to 1.8)	0.53 (−2.3 to 3.4)	
Momentary level of boredom^a^	−5.8 (−6.5 to −5.1)	**−5.2 (−5.8 to −4.5)**	1.0
Time of day			1.1
Morning	—	—	
Evening	1.3 (0.07 to 2.6)	0.31 (−0.88 to 1.5)	
Day of the week			1.0
Monday	—	—	
Tuesday	−1.3 (−3.8 to 1.2)	−0.79 (−3.0 to 1.5)	
Wednesday	−0.65 (−3.2 to 1.9)	0.02 (−2.3 to 2.3)	
Thursday	−1.1 (−3.6 to 1.4)	0.28 (−2.0 to 2.5)	
Friday	−0.29 (−2.9 to 2.3)	−0.50 (−2.9 to 1.9)	
Saturday	3.3 (0.72 to 5.8)	**2.4 (0.02 to 4.8)**	
Sunday	2.9 (−1.7 to 7.5)	1.6 (−2.6 to 5.8)	
Age (years)	0.54 (−1.8 to 2.9)	0.93 (−1.1 to 3.0)	1.0
Gender			1.0
Boy	—	—	
Girl	−7.5 (−12 to −2.8)	**−5.9 (−10 to −1.6)**	
Other	−21 (−36 to −7.3)	**−14 (−27 to −1.1)**	
Family socioeconomic status			1.0
Very advantaged	—	—	
Advantaged	−5.2 (−14 to 3.2)	−2.6 (−9.8 to 4.6)	
Intermediate	−6.7 (−15 to 2.0)	−4.0 (−11 to 3.5)	
Modest	−2.1 (−10 to 6.2)	−1.2 (−8.3 to 5.9)	
Does not know	−3.2 (−13 to 6.6)	−1.2 (−9.5 to 7.2)	
History of chronic health conditions			1.0
No	—	—	
Yes	−6.4 (−13 to 0.07)	−4.8 (−10 to 0.64)	

Abbreviations: GVIF = Generalized Variance Inflation Factor, CI = confidence interval.

Conditional R2: 0.689.

Marginal R2: 0.250.

Adjusted ICC: 0.585.

a Continuous variables were standardized prior to analysis.

b GVIFˆ[1/(2*df)].

No statistically significant association was observed between objective exposure to green space and momentary well-being (aB = 0.53, 95% CI: −0.30 to 1.4). However, there was a positive trend suggesting higher momentary well-being when adolescents were in a park at the time of questionnaire completion (aB = 5.8, 95%CI: −0.56 to 12). Days with more sunshine were associated with higher well-being (aB = 0.98, 95%CI: 0.21 to 1.7). We found no associations for community crime level (aB = 0.20, 95%CI: −1.3 to 0.89). However, while there was no relationship between adolescents’ own socioeconomic status and their momentary well-being, the socioeconomic deprivation of the area where adolescents were located at the time of questionnaire completion was associated with lower well-being (aB = −1.2, 95%CI: −2.2 to −0.17).

Compared to Mondays, adolescents experienced higher levels of momentary well-being on Saturdays (aB = 2.4, 95%CI: 0.02 to 4.8).

Finally, girls and gender-diverse adolescents reported significantly lower well-being than boys, even after adjusting for contextual characteristics of their immediate environment.

The marginal Nakagawa's R^2^ was 0.250, indicating that fixed effects accounted for approximately 25% of the variance in momentary well-being.

Results of sensitivity analyses (Supplementary Tables S4, S5, and S6) supported the robustness of the main findings. The observed associations remained unchanged after excluding gender-diverse participants and after accounting for school-level clustering. Removing boredom from the model yielded a similar pattern of results and further reinforced the positive association between social context and momentary well-being, with significant associations also observed when participants reported being with a single friend or parent.

## 4 Discussion

Our study advances current knowledge by applying an innovative GEMA approach to investigate momentary well-being among adolescents (i.e., a population underrepresented in GEMA research) in real-life contexts.

Our findings partially align with those observed in GEMA studies in adults. First, they highlight an important role of social relationships, with participants reporting higher well-being in the presence of several friends or family members. These results are consistent with a large body of research underscoring the importance of supportive and trusting social relationships in youth, to help adolescents overcome emotional difficulties and mitigate adverse mental health outcomes (Breedvelt et al., 2022). Our findings add a valuable temporal dimension, as this positive association is observable at a momentary level. In contrast, being among unknown people was associated with lower well-being. Studies with similar findings suggest that adolescents may be more inclined to repress their negative emotions when they are with strangers which may, in turn, negatively affect their mental health (Walsh et al., 2024). Estimates for other infrequent social contexts should be interpreted cautiously because of the limited number of observations.

Adolescents’ geographical environment was also associated with their momentary well-being. As in adults, sunlight was positively associated with well-being in our sample. Increased opportunity for physical activity and exposure to natural environments, as well as direct biological effects on neurotransmitter systems (Lambert et al., 2002) may underpin this finding. Future GEMA studies combining real-time measures of well-being, physical activity, and environmental exposures may help disentangle the behavioral and biological pathways linking sunlight exposure and mental health.

In contrast, and contrary to our hypotheses, we found no association between exposure to green space and momentary well-being. This finding should be interpreted in light of several methodological considerations. First, the final analytical sample may have provided sufficient power to detect relatively strong social associations while being underpowered to detect more modest environmental effects. Second, NDVI provides only a general measure of vegetation density and did not allow us to distinguish between potentially “virtuous” green spaces, such as parks or forests, and other types of vegetation, such as agricultural fields, which may be less beneficial for well-being. Previous studies have suggested that green space may contribute to mental health through indirect pathways (e.g., by enhancing opportunities for social interaction, promoting physical activity, or reducing harmful exposures to noise or air pollution (Markevych et al., 2017)), or through cumulative rather than immediate exposures (Yu et al., 2024). Further studies with larger samples and more refined measures of environmental exposure are needed to clarify the relationship between green space and momentary well-being among adolescents.

We observed that adolescents’ momentary well-being was associated with the socioeconomic status of the neighborhood in which they were located when they completed the questionnaire. Several mechanisms could explain this association, including differences in access to resources (Stafford & Marmot, 2003), environmental quality, or social conditions. However, these mechanisms were not measured directly in this study and therefore remain speculative.

Beyond contextual characteristics, our findings suggest that boredom is also associated with adolescents’ momentary well-being (Silk et al., 2011). Boredom is increasingly recognized as a modifiable determinant of adolescent health, as it has been identified as a risk factor for maladaptive behaviors, particularly when adolescents lack structured or supportive contexts, or when they experience depressive symptoms (Weybright et al., 2015; Baltacı and Açar).

Finally, girls and gender-diverse adolescents reported lower levels of momentary well-being than boys, consistent with findings from cross-sectional studies assessing well-being as a latent trait (Well-being and gender). Girls tend to experience higher levels of emotional distress when confronted with interpersonal stressors, which has been linked to greater exposure to appearance- and peer-related stress (Rudolph, 2002). Gender-diverse youth, on the other hand, tend to experience more psychosocial negative experiences, including stigma and lack of support, all of which are known contributors to chronic stress and reduced mental well-being (White et al., 2023). However, given the small number of gender-diverse adolescents in our sample, findings regarding this subgroup should be interpreted with caution.

Although causal inferences cannot be drawn because of our study design, the observed associations highlight several contextual factors that may warrant further investigation as potential targets for mental health promotion in adolescents. Future longitudinal or experimental studies could explore whether social connectedness, neighborhood characteristics, and environmental exposures contribute to adolescents' well-being through causal pathways amenable to intervention. In this context, Ecological Momentary Interventions (EMIs) may be promising (van Genugten et al., 2020). By leveraging real-time data, EMIs may provide one possible framework through which social connectedness, boredom and other contextual factors identified in this study could eventually be targeted (Kaufman et al., 2022).

Our study has several strengths. Assessment of momentary mental health relied on the EMoWI, a scale validated for the measurement of momentary well-being, which may enhance comparability of our findings with future studies using the same scale. Inclusion of both social and geographical variables allowed for broader understanding of adolescents’ momentary experiences. The consistency of findings across sensitivity analyses strengthens confidence in the robustness of the observed associations. Some additional social-context associations emerged when boredom was excluded from the model, which suggests that boredom may share variance with certain social-context variables, and provides additional support for the association between social context and momentary well-being.

However, some limitations must be noted. First, more than half of all questionnaires were excluded due to missing geolocation data. Reasons for missingness could not be determined from the available data, but may include technical issues, such as permission settings, or GPS deactivation on participants' smartphones. While no systematic differences were observed between excluded and included participants, we cannot exclude the possibility that missingness was related to unmeasured factors, such as participants' momentary activities, contexts, or emotional states. Consequently, geolocation data may not have been missing completely at random, and the final analytical sample may not fully represent either all GEMA participants or the broader EXIST cohort. In addition, the resulting reduction in sample size decreased statistical power limiting detection of associations between momentary well-being and exposure to green space or history of chronic health conditions, despite estimates suggestive of potential associations. Since geolocation data were essential for spatial modeling and were likely not missing at random, listwise deletion was considered the most appropriate and conservative approach. This limitation underscores the importance of providing participants with training prior to EMA data collection (Myin-Germeys & Kuppens, 2022), to guarantee technical requirements. Second, participation in the GEMA sub-study was voluntary, which could have introduced selection bias: compared to the general EXIST cohort, the GEMA sub-sample included a substantially lower proportion of boys and a higher proportion of adolescents from very advantaged socioeconomic backgrounds. These differences may reflect varying levels of interest or availability to participate in research and might limit the generalizability of our findings to the broader general adolescent population. Third, the relatively wide response window was chosen to maximize compliance among adolescents, and to increase the likelihood of capturing a range of everyday contexts, including commuting and other waiting periods that may contribute to adolescents' daily experiences. However, if the probability or delay of responding varied according to participants' momentary activities, social context or emotional state, some experiences may have been underrepresented, which should be considered when interpreting the findings. Fourth, the environmental indicators were derived from different spatial units and temporal scales, reflecting availability of existing data sources. While greenness (NDVI) and sunlight exposure reflected environmental conditions at the time and place of questionnaire completion, neighborhood deprivation and crime indicators were available only at broader administrative levels, and may not accurately reflect adolescents' immediate surroundings at the time of questionnaire completion. In particular, the FDEP index was derived from 2015 data, whereas EMA observations were collected in 2023–2024. Although socioeconomic deprivation reflects relatively stable structural characteristics of neighborhoods, some degree of temporal misclassification cannot be excluded. Fifth, because smartphone use is prohibited in most French secondary schools, our study primarily captured experiences occurring outside school hours. Given the central role of school in adolescents’ social interactions, daily activities, and emotional experiences, the underrepresentation of school-time observations may have influenced the distribution of social contexts and well-being levels observed in our study, and consequently, limited our ability to fully characterize the momentary associations between context and adolescent well-being. Sixth, the observational nature of this study precludes any causal interpretation of the associations identified. Although contextual characteristics and well-being were measured concurrently in real-life settings, the direction of these associations cannot be established. For example, while higher well-being was observed when adolescents were with friends or family members, it is equally possible that adolescents experiencing higher well-being were more likely to seek social interactions. Future studies using longitudinal designs are needed to clarify the directionality of these associations. Finally, several contextual factors examined are likely to co-occur in adolescents' daily lives. For example, being at home, spending time with family members, and weekend periods may represent overlapping aspects of the same social context. Although multicollinearity diagnostics did not indicate problematic statistical overlap, the observed associations should not be interpreted as fully distinct contextual influences. Rather, they reflect associations estimated after adjustment for other measured characteristics of the momentary environment.

## 5 Conclusion

Our study provides novel evidence on both the momentary experiences and individual and environmental characteristics associated with adolescents’ momentary well-being. Integrating these insights into public health strategies may help promote resilience and well-being in adolescents.

## CRediT authorship contribution statement

**Marie Buzzi:** Conceptualization, Methodology, Writing – original draft. **Nelly Agrinier:** Conceptualization, Methodology, Project administration, Supervision, Validation, Writing – review & editing. **Benoît Lalloué:** Data curation, Formal analysis, Methodology, Writing – review & editing. **Emeline Lequy:** Methodology, Writing – review & editing. **Fabienne Ligier:** Writing – review & editing. **Jennifer O'Loughlin:** Conceptualization, Writing – review & editing. **Yan Kestens:** Conceptualization, Methodology, Writing – review & editing. **Laetitia Minary:** Conceptualization, Data curation, Funding acquisition, Methodology, Project administration, Supervision, Validation, Writing – review & editing.

## Ethical statement

The EXIST cohort and the GEMA sub-study were approved by the *Comité éthique et scientifique pour les recherches, les études et les évaluations dans le domaine de la santé* (CESREES), the *Commission Nationale de l’Informatique et des Libertés* (CNIL) (n°922169), and the *Inserm* IRB, i.e., the *Comité d’Evaluation Ethique de l’Inserm* (CEEI) (n°21-837 and n°21-837 bis).

Prior to participating in EXIST, adolescents and their legal representatives were informed in writing about the study and their right to refuse to participate or to ask for removal of their data at any time.

## Funding

This research was funded by 10.13039/501100020320IReSP (Institut pour la recherche en santé publique) and 10.13039/501100006364INCa (Institut National du Cancer) as part of the call for research and intervention projects to reduce and combat smoking (Appel à projets de recherche et d'intervention pour réduire et lutter contre le tabagisme – Grant IRESP-19-TABAC-V1-02).

## Declaration of competing interest

The authors declare the following financial interests/personal relationships which may be considered as potential competing interests: Laetitia Minary reports financial support was provided by Institut pour la Recherche En Santé Publique (IReSP) and Institut National du Cancer (INCa). Yan Kestens reports a relationship with Polygon Research Inc. that includes: equity or stocks. If there are other authors, they declare that they have no known competing financial interests or personal relationships that could have appeared to influence the work reported in this paper.

## Data Availability

Data will be made available on request.

## List of abbreviations

EMA: Ecological Momentary Assessment
EMoWI: Ecological Momentary Wellbeing Instrument
FDEP: French Deprivation index
GEMA: Geographically-explicit Ecological Momentary Assessment
NDVI: Normalized Difference Vegetation Index
