# Supplementary material for: How do you feel right now? Investigating momentary well-being in French adolescents using geographically-explicit ecological momentary assessment
Source: SSM Popul Health. 2026 Aug 12;35:101956. doi: 10.1016/j.ssmph.2026.101956 (PMC13505490; doi:10.1016/j.ssmph.2026.101956)
Supplement: Supplementary file 1 [file mmc1.docx]

Supplementary Material

[Table S1. STROBE-GEMA checklist	2](#_Toc232071466)

[Table S2. Comparison of characteristics between participants included and excluded from spatial analyses	12](#_Toc232071467)

[Table S3. Comparison of characteristics between participants to the EXIST-GEMA sub-study and non-participants from the EXIST cohort	13](#_Toc232071468)

[Table S4. Results of sensitivity analysis excluding participants identifying outside the male–female binary	14](#_Toc232071469)

[Table S5. Results of sensitivity analysis including a school-level random effect	16](#_Toc232071470)

[Table S6. Results of sensitivity analysis excluding boredom from the adjusted model	18](#_Toc232071471)

[Table S7. Variance components and hierarchical structure of the primary analysis mixed-effects model	20](#_Toc232071472)

# Table S1. STROBE-GEMA checklist

| Item | Item # | STROBE Recommendations | Item # | Extension for GEMA studies (STROBE-GEMA) | Page |
| --- | --- | --- | --- | --- | --- |
| Title and abstract | | | | |  |
| Title and abstract | 1 | (a) Indicate the study’s design with a commonly used term in the title or the abstract  (b) Provide in the abstract an informative and balanced summary of what was done and what was found | 1 | (c) Include geographic ecological momentary assessment in the abstract and keywords | 1 |
| Background/  rationale | 2 | (a) Explain the scientific background and rationale for the investigation being reported | 2 | (b) Provide rationale for using GEMA for this study or topic of interest (e.g., to examine time and space variant/momentary environmental predictors (e.g., amount of greenness) of active daily mobility) | 3 |
| Objectives | 3 | State-specific objectives, including any prespecified hypotheses |  |  | 4 |
| Methods | | | | |  |
| Study design | 4 | Present key elements of study design early in the paper |  |  | 6-7 |
| Monitoring period |  |  | 4.1 | State the number of days each wave of the study lasted, and how many weekdays versus weekend days^a^ | 6 |
| Prompting design |  |  | 4.2 | (a) Describe prompting frequency (e.g. number of prompts per period of time) and associated rules (e.g., event based, geofencing trigger, schedule-based with random component, number of prompts per day, time available for completion)  (b) Describe prompt notification type when not self-triggered (e.g. app notification, text message)  (c) Describe prompting design for other variables of interest | 6-7 |
| Training |  |  | 4.3 | Indicate if, and how training of participants for GEMA protocol was conducted | 7 |
| Consent |  |  | 4.4 | a) Provide information about consent form and the ethical board certificate number  b) Indicate location precision data collection as per local ethical committee ruling | 10 |
| Setting | 5 | Describe the setting, locations, and relevant dates, including periods of recruitment, exposure, follow-up, and data collection |  |  | 6-7 |
| Technology |  |  | 6 | (a) Describe what technology was used, both for the momentary questionnaires (e.g. name of the application used for EMA prompts) and for capturing geographic location (e.g. mobile phone with or without separate GPS receiver, assisted GPS, laptop, wifi-based, self-report map-based question) and specify if the technology was provided to the participants or if they used their own device (e.g. cellphone)  (b) Report any supplementary devices or embedded sensors used to measure other variables of interest (e.g. light, noise. physical activity) and provide relevant information regarding their use  (c) Indicate if the technology used real-time or delayed data transmission  (d) Indicate rules for technology use if any (e.g. GPS-tracker to be worn at the hip at all times, mobile phone to be carried at all times, etc.)  (e) Indicate what app or web-app was used (if applicable) for data collection  (f) Indicate if the technology used required recharging | 6-7 |
| Participants | 7 | **(a) *Cohort study***—Give the eligibility criteria, and the sources and methods of selection of participants. Describe methods of follow-up  ***Case-control study***—Give the eligibility criteria, and the sources and methods of case ascertainment and control selection. Give the rationale for the choice of cases and controls  ***Cross-sectional study***—Give the eligibility criteria, and the sources and methods of selection of participants  **(b) *Cohort study***—For matched studies, give matching criteria and number of exposed and unexposed  ***Case-control study***—For matched studies, give matching criteria and the number of controls per case |  |  | 6 |
| Variables | 8 | (a) Clearly define all outcomes, exposures, predictors, potential confounders, and effect modifiers. Give diagnostic criteria, if applicable | 8 | (b) Provide information for variables derived from EMA questionnaires, and GPS or other location variables available that were used (Lat/Long; number of visible satellites, indoor/outdoor indicator)  (c) Clearly define all environmental exposure variables  (d) Provide temporal and spatial resolution of main EMA outcomes and covariates | 7-9 |
| Data sources/ measurement | 9 | (a) For each variable of interest, give sources of data and details of methods of assessment (measurement). Describe the comparability of assessment methods if there is more than one group | 9 | (b) Describe what geographic coordinates and metadata were available (e.g. Latitude/Longitude, number of visible satellites, HDOP/PDOP, indoor/outdoor indicator)  (c) Describe how location coordinates were used to derive momentary or cumulative environmental exposure(s) (e.g. distance to exposure, density, use of buffers)  (d) Describe temporal relation between EMA questions answered and location data (e.g. simultaneous punctual, cumulative location tracking during X minutes previous to answer to prompt)  (e) Describe measurement instruments for all EMA questions, and if the instrument has been validated in an EMA context.  (f) Describe possible distribution of instruments through prompts (i.e. what questions came with what prompt)  (g) Specify the exact source(s) of location data (self-reported, device-based passive data collection, combination of both) | 7-9 |
| Bias / Design features | 10 | (a) Describe any efforts to address potential sources of bias | 10 | (b) Describe any design feature to address potential sources of bias (eg, reactivity) or participant burden (eg, EMA questions appearing in different orders) or spatial bias  (including mitigation efforts to reduce potential selective daily mobility bias, the collection of widely-varied amounts of geo-data from individuals (e.g., those that move around more) and then running analyses that do not properly weight for such within and between person sources of geographically-specific sampling bias, accuracy of location variables) | N/A |
| Study size | 11 | Explain how the study size was arrived at | 11 | (a) Explain how the study size was arrived at, both in terms of number of participants and number of surveys | 10-11 |
| 10-11 Quantitative variables | 12 | Explain how quantitative variables were handled in the analyses. If applicable, describe which groupings were chosen and why |  |  | 9 |
| Statistical methods (model) | 13 | (a) Describe all statistical methods, including those used to control for confounding  (b) Describe any methods used to examine subgroups and interactions  (c) Explain how missing data were addressed  (d) *Cohort study*—If applicable, explain how the loss to follow-up was addressed  *Case-control study*—If applicable, explain how the matching of cases and controls was addressed  *Cross-sectional study*—If applicable, describe analytical methods taking account of the sampling strategy  (e) Describe any sensitivity analyses | 13 | (f) Indicate the number of levels of analysis  (e.g., whether the statistical unit of analysis was the prompt, the day, or the individual)  (g) Clearly explain and justify the number of levels and nesting structure used if multilevel analysis was used as often recommended (e.g. prompts within days within individuals) | 9 |
| Results | | | | |  |
| Participants / Attrition | 14 | (a) Report numbers of individuals at each stage of study—e.g., numbers potentially eligible, examined for eligibility, confirmed eligible, included in the study, completing follow-up, and analyzed  (b) Give reasons for non-participation at each stage  (c) Consider the use of a flow diagram | 14 | (d) Define what is valid data and indicate the proportion of invalid data  (e) Absence/Presence of GPS coordinate when momentary questionnaire is answered  (f) Indicate participant attrition throughout the study; report attrition rates both by monitoring days and waves, if applicable^a^ | 11 |
| Prompt delivery and completion^a^ |  |  | 15 | (a) Report number of EMA prompts that were planned to be delivered. If possible, also report the number of EMA prompts that were actually received by participants and indicate reasons for why prompts were not sent out (eg, technical issues or participant noncompliance reason such as phone was powered off)^a^ | 11 |
| Latency^a^ |  |  | 16 | Report the amount of time from prompt signal to answering of prompt^a^ | Non available |
| Compliance rate^a^ |  |  | 17 | (a) Report total answered EMA prompts across all subjects and the average number of EMA prompts answered per person. Report compliance rate both by monitoring days, time of day and waves, if applicable. Indicate reasons for noncompliance, if known^a^  (b) Report proportion of participants who did not allow location data capture, if available | 11 |
| Descriptive data | 18 | (a) Give characteristics of study participants (e.g. demographic, clinical, social) and information on exposures and potential confounders  (b) Indicate the number of participants with missing data for each variable of interest  (c) *Cohort study*—Summarize follow-up time (e.g., average, and total amount) |  |  | 11-12 |
| Missing data^a^ |  |  | 19 | (a) Report whether EMA compliance is related to demographic or time-varying variables^a^  (b) Report proportion of answered prompts with and without location data (e.g. missing GPS coordinates), and whether missing GPS data is related to demographic, time-varying variables or hardware/OS used if available (e.g. smartphone model, OS version) | 12 |
| Outcome data | 20 | *Cohort study*—Report numbers of outcome events or summary measures over time  *Case-control study—*Report numbers in each exposure category, or summary measures of exposure  *Cross-sectional study—*Report numbers of outcome events or summary measures |  |  | 14-15 |
| Main results | 21 | (a) Give unadjusted estimates and, if applicable, confounder-adjusted estimates and their precision (eg, 95% confidence interval). Make clear which confounders were adjusted for and why they were included  (b) Report category boundaries when continuous variables were categorized  (c) If relevant, consider translating estimates of relative risk into absolute risk for a meaningful time period |  |  | 14-15 |
| Other analyses | 22 | Report other analyses done—eg analyses of subgroups and interactions, and sensitivity analyses |  |  | N/A |
| Discussion | | | | |  |
| Key results | 23 | Summarize key results with reference to study objectives |  |  | 15-16 |
| Limitations | 24 | Discuss the limitations of the study, taking into account sources of potential bias or imprecision. Discuss both the direction and magnitude of any potential bias |  | (a) Discuss limitations of the study, taking into account sources of potential bias when using GEMA methods (e.g., reactivity, use of technology, sample selection bias due non answered EMA prompt or missing geographic location) | 17 |
| Interpretation / Conclusions | 25 | a) Give a cautious overall interpretation of results considering objectives, limitations, multiplicity of analyses, results from similar studies, and other relevant evidence |  | b) Discuss whether and how GEMA was relevant to address the research question | 17-18 |
| Generalizability | 26 | Discuss the generalizability (external validity) of the study results |  |  | 17 |
| Other information | | | | |  |
| Funding | 27 | Give the source of funding and the role of the funders for the present study and, if applicable, for the original study on which the present article is based |  |  | 23 |

# Table S2. Comparison of characteristics between participants included and excluded from spatial analyses

|  | Excluded N = 108 | Included N = 122 |
| --- | --- | --- |
| Age |  |  |
| Median (Q1 - Q3) | 13.42 (13.17 - 13.71) | 13.42 (13.25 - 13.63) |
| Gender |  |  |
| Boy | 31 (28.7%) | 42 (34.4%) |
| Girl | 77 (71.3%) | 77 (63.1%) |
| Other | 0 (0%) | 3 (2.5%) |
| Socioeconomic status |  |  |
| Very advantaged | 12 (11.1%) | 14 (11.5%) |
| Advantaged | 27 (25%) | 32 (26.2%) |
| Intermediate | 18 (16.7%) | 26 (21.3%) |
| Modest | 34 (31.4%) | 36 (29.5%) |
| Disadvantaged | 1 (0.9%) | 0 (0%) |
| Does not know | 16 (14.8%) | 14 (11.5%) |
| Pre-existing health issue |  |  |
| No | 92 (85.2%) | 102 (83.6%) |
| Yes | 16 (14.8%) | 20 (16.4%) |

# Table S3. Comparison of characteristics between participants to the EXIST-GEMA sub-study and non-participants from the EXIST cohort

|  | Non-participants  N = 5,264 | Participants to the GEMA sub-study N = 230 | Total  N = 5,494 | p-value*^1^* |
| --- | --- | --- | --- | --- |
| Age |  |  |  | 0.020 |
| Median (Q1 - Q3) | 13.5 (13.2 - 13.8) | 13.4 (13.2 - 13.7) | 13.5 (13.2 - 13.8) |  |
| *Missing* | 11 | 0 | 11 |  |
| Gender |  |  |  | **<0.001** |
| Boy | 2,694 (51%) | 73 (32%) | 2,767 (50%) |  |
| Girl | 2,511 (48%) | 154 (67%) | 2,665 (48%) |  |
| Other | 56 (1.1%) | 3 (1.3%) | 59 (1.1%) |  |
| *Missing* | 3 | 0 | 3 |  |
| Familial socioeconomic status |  |  |  | **<0.001** |
| Very advantaged | 337 (6.4%) | 26 (11%) | 363 (6.6%) |  |
| Advantaged | 1,156 (22%) | 59 (26%) | 1,215 (22%) |  |
| Intermediate | 840 (16%) | 44 (19%) | 884 (16%) |  |
| Modest | 1,617 (31%) | 70 (30%) | 1,687 (31%) |  |
| Disadvantaged | 89 (1.7%) | 1 (0.4%) | 90 (1.6%) |  |
| Does not know | 1,225 (23%) | 30 (13%) | 1,255 (23%) |  |
| History of chronic health condition |  |  |  | 0.19 |
| No | 4,560 (87%) | 194 (84%) | 4,754 (87%) |  |
| Yes | 656 (13%) | 36 (16%) | 692 (13%) |  |
| *Missing* | 48 | 0 | 48 |  |
| ^1^ *Wilcoxon-Mann-Whitney test; Fisher’s Exact Test for Count Data with simulated p-value (based on 2000 replicates); exact Fisher’s test* | | | | |

# Table S4. Results of sensitivity analysis excluding participants identifying outside the male–female binary

| Characteristic (N=1,229) | Adjusted model | Adjusted GVIF^1^ |
| --- | --- | --- |
|  | **Beta (95% CI)** |  |
| Where are you? |  | 1.1 |
| At home | - |  |
| At school | -0.81 (-4.3 to 2.7) |  |
| Traveling (e.g., at a bus stop, on a train, in a car, etc.) | -0.95 (-2.9 to 0.97) |  |
| At a sports or leisure facility | 0.29 (-3.7 to 4.3) |  |
| In a park | 5.8 (-0.49 to 12) |  |
| At a friend’s house | **6.1 (0.44 to 12)** |  |
| At a party | -0.94 (-7.3 to 5.4) |  |
| Other | -0.48 (-3.0 to 2.0) |  |
| Average NDVI within a 100m radius* | 0.51 (-0.33 to 1.3) | 1.1 |
| French Deprivation Index* | **-1.1 (-2.1 to -0.12)** | 1.0 |
| Community crime level (‰)* | -0.21 (-1.3 to 0.89) | 1.1 |
| Sunshine duration on measurement day (hours)* | **0.91 (0.13 to 1.7)** | 1.1 |
| Who are you with right now? |  |  |
| *A friend* |  | 1.1 |
| No | - |  |
| Yes | 0.80 (-1.5 to 3.1) |  |
| *Several friends* |  | 1.2 |
| No | - |  |
| Yes | **3.2 (0.78 to 5.7)** |  |
| *My father or my mother* |  | 1.0 |
| No | - |  |
| Yes | 0.95 (-0.44 to 2.3) |  |
| *One or several family members* |  | 1.1 |
| No | - |  |
| Yes | **2.8 (1.3 to 4.2)** |  |
| *Persons I do not know* |  | 1.1 |
| No | - |  |
| Yes | **-4.4 (-7.5 to -1.3)** |  |
| *Other* |  | 1.0 |
| No | - |  |
| Yes | 0.34 (-2.6 to 3.2) |  |
| Momentary level of boredom* | **-5.2 (-5.9 to -4.5)** | 1.0 |
| Time of day |  | 1.1 |
| Morning | - |  |
| Evening | 0.25 (-0.95 to 1.5) |  |
| Day of the week |  | 1.0 |
| Monday | - |  |
| Tuesday | -0.71 (-3.0 to 1.6) |  |
| Wednesday | 0.09 (-2.2 to 2.4) |  |
| Thursday | 0.32 (-2.0 to 2.6) |  |
| Friday | -0.61 (-3.0 to 1.8) |  |
| Saturday | **2.4 (0.04 to 4.8)** |  |
| Sunday | 1.6 (-2.7 to 6.0) |  |
| Age (years) | 0.98 (-1.0 to 3.0) | 1.0 |
| Gender |  |  |
| Boy | - |  |
| Girl | **-6.0 (-10 to -1.7)** |  |
| Family socioeconomic status |  | 1.0 |
| Very advantaged | - |  |
| Advantaged | -2.4 (-9.6 to 4.8) |  |
| Intermediate | -3.6 (-11 to 3.9) |  |
| Modest | -1.6 (-8.7 to 5.5) |  |
| Does not know | -1.2 (-9.5 to 7.1) |  |
| History of chronic health conditions |  | 1.0 |
| No | - |  |
| Yes | -4.3 (-9.9 to 1.3) |  |
| **Continuous variables were standardized prior to analysis*  *^1^ GVIF^[1/(2*df)]*  *Abbreviations: GVIF = Generalized Variance Inflation Factor, CI = confidence interval*  Conditional R2: 0.684  Marginal R2: 0.228  Adjusted ICC: 0.591 | | |

# Table S5. Results of sensitivity analysis including a school-level random effect

| Characteristic (N=1,229) | Adjusted model | Adjusted GVIF^1^ |
| --- | --- | --- |
|  | **Beta (95% CI)** |  |
| Where are you? |  | 1.1 |
| At home | - |  |
| At school | -0.91 (-4.4 to 2.5) |  |
| Traveling (e.g., at a bus stop, on a train, in a car, etc.) | -0.97 (-2.9 to 0.91) |  |
| At a sports or leisure facility | 0.66 (-3.3 to 4.6) |  |
| In a park | 5.8 (-0.56 to 12) |  |
| At a friend’s house | **5.9 (0.25 to 12)** |  |
| At a party | 0.25 (-5.8 to 6.3) |  |
| Other | -0.43 (-2.9 to 2.1) |  |
| Average NDVI within a 100m radius* | 0.53 (-0.30 to 1.4) | 1.1 |
| French Deprivation Index* | **-1.2 (-2.2 to -0.17)** | 1.0 |
| Community crime level (‰)* | -0.20 (-1.3 to 0.89) | 1.1 |
| Sunshine duration on measurement day (hours)* | **0.98 (0.21 to 1.7)** | 1.1 |
| Who are you with right now? |  |  |
| *A friend* |  | 1.1 |
| No | - |  |
| Yes | 1.2 (-1.0 to 3.4) |  |
| *Several friends* |  | 1.2 |
| No | - |  |
| Yes | **3.5 (1.1 to 5.9)** |  |
| *My father or my mother* |  | 1.0 |
| No | - |  |
| Yes | 1.0 (-0.38 to 2.4) |  |
| *One or several family members* |  | 1.1 |
| No | - |  |
| Yes | **3.0 (1.6 to 4.5)** |  |
| *Persons I do not know* |  | 1.1 |
| No | - |  |
| Yes | **-4.6 (-7.7 to -1.5)** |  |
| *Other* |  | 1.0 |
| No | - |  |
| Yes | 0.53 (-2.3 to 3.4) |  |
| Momentary level of boredom* | **-5.2 (-5.8 to -4.5)** | 1.0 |
| Time of day |  | 1.1 |
| Morning | - |  |
| Evening | 0.31 (-0.88 to 1.5) |  |
| Day of the week |  | 1.0 |
| Monday | - |  |
| Tuesday | -0.79 (-3.0 to 1.5) |  |
| Wednesday | 0.02 (-2.3 to 2.3) |  |
| Thursday | 0.28 (-2.0 to 2.5) |  |
| Friday | -0.50 (-2.9 to 1.9) |  |
| Saturday | **2.4 (0.02 to 4.8)** |  |
| Sunday | 1.6 (-2.6 to 5.8) |  |
| Age (years) | 0.93 (-1.1 to 3.0) | 1.0 |
| Gender |  | 1.0 |
| Boy | - |  |
| Girl | **-5.9 (-10 to -1.6)** |  |
| Other | **-14 (-27 to -1.1)** |  |
| Family socioeconomic status |  | 1.0 |
| Very advantaged | - |  |
| Advantaged | -2.6 (-9.8 to 4.6) |  |
| Intermediate | -4.0 (-11 to 3.5) |  |
| Modest | -1.2 (-8.3 to 5.9) |  |
| Does not know | -1.2 (-9.5 to 7.2) |  |
| History of chronic health conditions |  | 1.0 |
| No | - |  |
| Yes | -4.8 (-10 to 0.64) |  |
| **Continuous variables were standardized prior to analysis*  *^1^ GVIF^[1/(2*df)]*  *Abbreviations: GVIF = Generalized Variance Inflation Factor, CI = confidence interval*  Conditional R2: NA  Marginal R2: 0.446 | | |

# Table S6. Results of sensitivity analysis excluding boredom from the adjusted model

| Characteristic (N=1,229) | Adjusted model | Adjusted GVIF^1^ |
| --- | --- | --- |
|  | **Beta (95% CI)** |  |
| Where are you? |  | 1.1 |
| At home | - |  |
| At school | -2.4 (-6.1 to 1.3) |  |
| Traveling (e.g., at a bus stop, on a train, in a car, etc.) | -1.3 (-3.4 to 0.70) |  |
| At a sports or leisure facility | 0.40 (-3.8 to 4.7) |  |
| In a park | 6.8 (-0.01 to 14) |  |
| At a friend’s house | **8.1 (2.0 to 14)** |  |
| At a party | 1.3 (-5.2 to 7.9) |  |
| Other | 0.08 (-2.6 to 2.8) |  |
| Average NDVI within a 100m radius* | 0.41 (-0.49 to 1.3) | 1.1 |
| French Deprivation Index* | **-1.3 (-2.4 to -0.19)** | 1.0 |
| Community crime level (‰)* | -0.86 (-2.0 to 0.32) | 1.1 |
| Sunshine duration on measurement day (hours)* | **0.95 (0.11 to 1.8)** | 1.1 |
| Who are you with right now? |  |  |
| *A friend* |  | 1.1 |
| No | - |  |
| Yes | **2.7 (0.28 to 5.1)** |  |
| *Several friends* |  | 1.2 |
| No | - |  |
| Yes | **5.5 (2.9 to 8.1)** |  |
| *My father or my mother* |  | 1.0 |
| No | - |  |
| Yes | **1.5 (0.01 to 3.0)** |  |
| *One or several family members* |  | 1.1 |
| No | - |  |
| Yes | **4.0 (2.4 to 5.6)** |  |
| *Persons I do not know* |  | 1.1 |
| No | - |  |
| Yes | **-5.5 (-8.8 to -2.1)** |  |
| *Other* |  | 1.0 |
| No | - |  |
| Yes | 0.02 (-3.1 to 3.1) |  |
| Time of day |  | 1.1 |
| Morning | - |  |
| Evening | 0.84 (-0.44 to 2.1) |  |
| Day of the week |  | 1.0 |
| Monday | - |  |
| Tuesday | -0.63 (-3.1 to 1.8) |  |
| Wednesday | 0.12 (-2.4 to 2.6) |  |
| Thursday | -0.41 (-2.9 to 2.0) |  |
| Friday | -0.25 (-2.8 to 2.3) |  |
| Saturday | **3.1 (0.53 to 5.7)** |  |
| Sunday | 1.1 (-3.5 to 5.7) |  |
| Age (years) | 0.51 (-1.8 to 2.8) | 1.0 |
| Gender |  | 1.0 |
| Boy | - |  |
| Girl | **-7.2 (-12 to -2.4)** |  |
| Other | **-21 (-35 to -6.3)** |  |
| Family socioeconomic status |  | 1.0 |
| Very advantaged | - |  |
| Advantaged | -3.5 (-12 to 4.6) |  |
| Intermediate | -4.8 (-13 to 3.6) |  |
| Modest | -2.2 (-10 to 5.8) |  |
| Does not know | -2.1 (-11 to 7.3) |  |
| History of chronic health conditions |  | 1.0 |
| No | - |  |
| Yes | -6.1 (-12 to 0.08) |  |
| **Continuous variables were standardized prior to analysis*  *^1^ GVIF^[1/(2*df)]*  *Abbreviations: GVIF = Generalized Variance Inflation Factor, CI = confidence interval*  Conditional R2: 0.676  Marginal R2: 0.149  Adjusted ICC: 0.619 | | |

# Table S7. Variance components and hierarchical structure of the primary analysis mixed-effects model

| **Level** | **Number of observations** | **Variance** | **Proportion of total variance (%)** |
| --- | --- | --- | --- |
| Day | 902 | 14.79 | 7.6% |
| Participant | 122 | 99.54 | 50.9% |
| Residual | - | 80.99 | 41.5% |
